# Imitation as Faithful Copying of a Novel Technique in Marmoset Monkeys

**DOI:** 10.1371/journal.pone.0000611

**Published:** 2007-07-11

**Authors:** Bernhard Voelkl, Ludwig Huber

**Affiliations:** Department for Neurobiology and Cognition Research, University of Vienna, Vienna, Austria; Università di Parma, Italy

## Abstract

Imitative learning has received great attention due to its supposed role in the development of culture and the cognitive demands it poses on the individual. Evidence for imitation in non-human primate species, therefore, could shed light on the early origins of proto-cultural traits in the primate order. Imitation has been defined as the learning of an act by seeing it done or, more specifically, as the copying of a novel or otherwise improbable act. But despite a century of research and the detection of mirror neurons the empirical basis for this most advanced form of observational learning is weak. Few, if any, studies have shown that the observer has learned the response topography, i.e., the specific action by which the response is made. In an experimental set-up we confronted marmoset monkeys (*Callithrix jacchus*) with a conspecific model that was previously trained to open a plastic box in a peculiar way. Employing detailed motion analyses we show that the observers precisely copied the movement patterns of the novel action demonstrated by the model. A discriminant analysis classified 13 out of 14 observer movements (92.86%) as model movements and only one as non-observer movement. This evidence of imitation in non-human primates questions the dominant opinion that imitation is a human-specific ability. Furthermore, the high matching degree suggests that marmosets possess the neuronal mechanism to code the actions of others and to map them onto their own motor repertoire, rather than priming existing motor-templates.

## Introduction

One of the best ways to learn how to execute a given action is by observing someone else performing it first. For example, most of us who have ever tried to play golf or tennis will have had a strong preference to learn by watching a professional instructor than by solely relying on a manual. Likewise in monkeys learning how to open encapsulated fruits may be learned most efficiently by imitating experienced conspecifics [1]. Imitation has been defined as the learning of an act by seeing it done [2] or, more specifically, as the copying of a novel or otherwise improbable act [3]. Despite a century of research, however, the empirical basis for this “cheap trick” is weak [4]. Few, if any, studies have shown that the observer has learned the response topography, i.e., the specific action by which the response is made [5]–[7]. As a first step, we showed that marmosets are capable of imitating the overall feature of the opening action, that is, of using the same body part as the model to open a food container [8]. Now we have aimed at the crucial step by quantitatively assessing the matching degree between the actions of the model and the observers.

A positive result has implications for two core problems of imitation research, the ‘transfer of skill’ [9] and the correspondence problem [10], [11]. It would suggest that not only humans and great apes but also monkeys have the potential to acquire novel behaviors by observation, thereby laying the foundation for cultural transmission [12], [13]. It would also force the currently available theories of imitation to explain how a visual representation of a new action can be transformed into motor output without relying on already existing behavior [14], [15]. Finally, we would have found the missing link of monkey social cognition, specifically between their possession of mirror neurons [16] and some recently proved capacities in the periphery of true imitation. The latter include copying of an expert's use of a rule [17], recognizing when being imitated [18], and replicating adult facial movements as neonates [19]. Positive results would corroborate earlier claims that marmoset monkeys are able to imitate [8], and furthermore would suggest that they need not rely on existing motor templates but can translate the actions of others directly into motor output.

## Results

**Figure 1:**
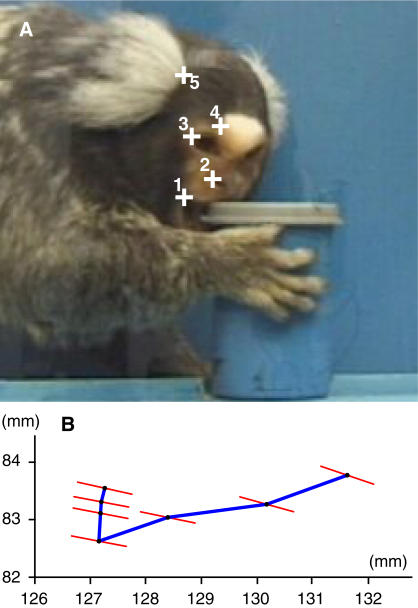
Motion analysis of the opening movement. (A) Five morphological features were identified as trace-points: (1) corner of the mouth, (2) outer corner of the nostril, (3) canthus, (4) corner of the white spot of the forehead, (5) a corner at the base of the ear-tufts. (B) Representation of the model's head position in 1/25 s time intervals: black dots represent the center of gravity of the trace-points, while thin red lines indicate head inclination.

**Figure 2:**
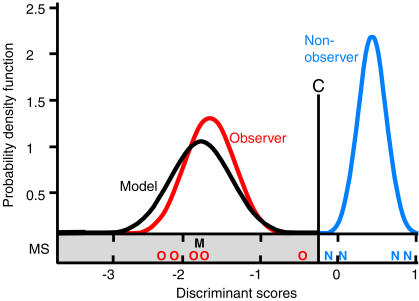
Probability density function for the mean discriminant scores. C: discrimination criterion; individual scores smaller than the criterion were classified as model scores (gray area) and larger scores were classified as non-observer scores. MS: mean scores of the observers (O), non-observers (N) and the model (M).

**Figure 3:**
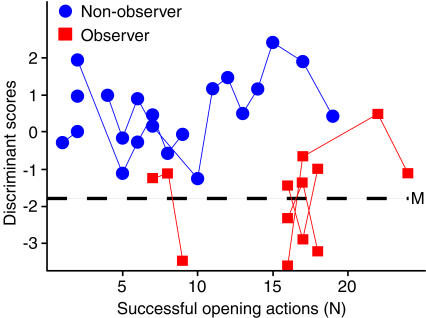
Relationship between discriminant scores (as measure for similarity) and prior experience of the subjects. Discriminant scores for all individual actions are plotted as a function of the number of successfully mouth-opened (completely closed) canisters prior to the movement that was analyzed. M: mean discriminant score of the model. Actions by the same individuals are connected by solid lines.

To investigate imitative learning in the sense of precisely copying an observed action, we compared the actions executed by observers with those of a model and, as a control, with those of non-observers, i.e. conspecifics that haven't seen the action before. In a previous experiment [8], one animal used a peculiar technique to open baited film canisters: instead of opening the canisters by hand, as most of the control subjects did, it used its mouth. Six subjects (observers) were then allowed to observe this model before they were confronted with the canister individually. Now we analyzed the opening movements (Figure 1) of these observers and compared them with those of the model and 24 naïve animals (non-observers). Five out of six observers but only four out of 24 non-observers succeeded in opening the canisters with their mouth. The nine successful animals were all adult females. We examined the successful opening movements of completely closed canisters of the model, the observers and the non-observers. The head movements of the subjects were tracked by manually identifying the position of five morphological features in the subject's face on a frame-to-frame basis (25 frames per s). We used five parameters to describe the movement: the change in the inclination of the head during the opening action, the overall direction of the movement, the total path length and the direct path length of the movement, and a detour factor defined as the fraction of the total path length divided by the direct path length. These parameters varied considerably with the total range for successful opening movements being on average three (3.1±1.8) times as large as the range for the successful opening movements of the model alone. As this demonstrates that the path to successful opening is rather broad, similarities between movement patterns of model and observers cannot be explained by functional constraints alone. A discriminant function analysis (DFA) of the orthogonalized data produced a function with clearly distinctive mean discriminant scores for movements of the model and the non-observers. To account for dependencies due to repeated sampling of the same individuals and unequal group-sizes, we employed a hierarchical bootstrap procedure (with 10,000 repetitions) to estimate mean discriminant scores. The mean discriminant scores for the observers were closer to the mean of the model than to the non-observer in 99.96% of the cases, and were within the 95 percentile range of the model in 96.61%; the scores were never within the 95 percentile range of the non-observers (Figure 2). Using the discriminant scores of the individual observer movements, we classified 13 out of 14 observer movements (92.86%) as model movements and only one as non-observer movement (Binomial test, two tailed, p = 0.002). The two components of the principal components analysis that had eigenvalues greater than 1.0 were highly correlated with four of the five motion parameters, and the third component with an eigenvalue marginally below one (0.98) was correlated with the fifth motion parameter. Hence, we cannot ascribe the discriminative power of the DFA to a single motion parameter, but to the combination of parameters, i.e. to the overall pattern of the movement. These general movement patterns of the observers were more similar to the movement pattern of the model than to those of the non-observers. In order to examine whether the movements converged towards the model pattern through practice, we plotted the discriminant scores against the number of successfully mouth-opened (completely closed) canisters (Figure 3). Neither observer nor non-observer movements show a trend towards increasing correspondence with the model movement.

## Discussion

The ability of marmosets to create exact replica of a technique for opening a plastic box demonstrated by a skilled conspecific model offers answers to two prevalent questions in imitation research. First, it demonstrates that monkeys are capable of learning new skills by imitation. The prevalent assumption of primatologists and developmental psychologists is that monkeys are only capable of simpler forms of social learning such as response facilitation, socially aided trial and error learning, and stimulus enhancement [20]–[22]. The present data, however, provide compelling evidence that action imitation is not an ability restricted to humans or the great apes, but that it has a much longer evolutionary record [23], [24]. Second, it provides evidence that monkeys possess a neuronal mechanism for directly transforming a visual representation of an action into motor output or can adjust existent representations of motor acts [25]. This clearly challenges those theories of imitation for which the key to solution is activation of *existing* motor representations.

Generalist or associative models would have no problems to include marmosets into the range of species capable of imitation, because they rely solely on task- and species-general processes of associative learning and action control. For instance, the ‘associative sequence learning’ (ASL) model [10], [26] explains the imitative capacity in terms of learned perceptual-motor links (contiguity-based ‘matching vertical associations’) of action units that become sequentially combined by action observation. Marmosets might have performed movements similar to the copied mouth action before, like biting into an object or levering it up with the head. The opening movement of the model can be described best as one short, smooth, and slightly U-shaped for- and upwards movement with a quite constant head inclination. Thus, it seems unlikely that the model's idiosyncratic opening technique can be decomposed into discrete, already stored action units, which then become integrated in a new manner by observing the model.

Specialist or transformational theories suggest that the correspondence problem is solved by activating an innate, species-specific cognitive mechanism that represents observed actions in a special-purpose ‘supramodal’ or symbolic code [27]. These theories would also require the observers to have had a motor representation of the model's film-canister-opening action before they observed the model. This code would subsequently be activated in the course of action-perception coupling. Based on the significant difference in the movement shown by observers and non-observers, however, opening a film box is not an all-or-nothing behavior for marmosets. There are still many degrees of freedom for the exact performance, created by the movements of the head and the whole body when attempting to open the lid of the film canister. Hence, explaining the data along the lines of a transformational theory requires assuming that all possible movement programs had been present in the observers' brain and that, by observing the model's action, the matching motor representation was selected. Making even the extremely simplistic assumption that every muscle can have just two states (contracted or relaxed – a clearly insufficient assumption here) means that 4×10^180^ motor programs would be necessary to yield a complete set of simple movement patterns to choose from [28]. This exceeds both the memory capacity and computation time of any matching mechanism.

Mirror neurons also cannot provide a straightforward solution to imitative learning. These neurons in the macaque's premotor cortex code the likely future actions of others so that observers can anticipate their behaviour, but they do not code the details of observed behavior [29]. Although so-called *tool-responding mirror neurons* with experience-dependent responses have been found, they discharge only after a relatively long visual exposure to actions of a tool-using experimenter; this suggests a functional role for motor training only [30].

Recently, a circuitry composed of the STS, the rostral sector of the inferior parietal lobule, and the ventral premotor cortex (area F5) has been suggested to code the action of others and to map it onto the observer's motor repertoire [31]. Imitative learning may be implemented by interactions among the core imitation circuit, the dorsolateral prefrontal cortex (BA46) and a set of areas relevant to motor preparation. This model fits into a conceptual framework for motor learning and sensorimotor control, the MOSAIC (Modular Selection And Identification for Control) architecture. This architecture is based on multiple pairs of ‘predictor’ and ‘controller’ models that process feedforward and feedbackward sensorimotor information, respectively [28]. To resolve seemingly contradictory results of imitation studies, a dual route theory of imitation was forwarded that assumes two distinctive types of imitation [32]. The first is the imitation of actions for which the observer can identify a goal and possesses a template in the long-term memory; this activates the supplementary motor area, the orbitofrontal cortex and the left inferior parietal lobule. The second is the imitation of novel actions whose goal can only be identified retrospectively; this takes a direct route, bypassing long-term memory and transforming visuo-spatial characteristics directly into motor representations.

The present findings that monkeys can learn novel actions by imitation suggest that they can use the direct imitation route. The existence of mirror neurons that help detect the goal of another's action, and that can recognize when they are being imitated, suggests that monkeys are potentially capable of utilizing both routes to imitation, although this issue requires further experimentation.

## Materials and Methods

### Subjects

Subjects were 31 adult common marmosets (*Callithrix jacchus*), maintained in seven family groups in indoor/outdoor cages measuring between 15 m^3^ and 30 m^3^. All animals were born in captivity. All cages were equipped with branches, ropes and enrichment devices. The animals were fed a mixed diet of fruits, vegetables, monkey pellets, insects and protein supplements. For the experiments the animals were let into a 60 cm×40 cm×120 cm experimental cage. The animals entered this experimental cage voluntarily through a path-way system. All animals were habituated to the experimental cage, the path-way system, the experimental routine and the experimenter. Housing conditions and experimental protocol were in accordance with Austrian legal regulations and the guidelines for the treatment of animals in behavioral research and teaching of the Association for the Study of Animal Behavior (ASAB).

The observer group consisted of five females and one male, and the non-observer group consisted of 18 females and six males. The number of subjects in the non-observer group exceeded the one of the observer group four-fold. These unequal group-sizes were necessary because most of the non-observers did not succeed in completing the task and could, therefore, not be used for the motion analysis. The subjects that participated in the preceding study [8] had no experience with the plastic canisters that were used in that study prior to that experiment. During that study they were confronted with 15 half shut canisters, and 15 completely shut canisters of which they opened between 0 and 15 with their mouth. This study was conducted directly thereafter and the subjects had no additional contact with the plastic canisters between the two studies. The subjects used as non-observers had been given between 6 and 14 half shut canister but no completely shut canisters prior to this experiment. However, as there was a gap of more than half a year between the subjects' first encounter of half shut canisters and the start of this experiment we decided to accustom them to the canisters anew (see below).

### Procedure

The plastic box was a black plastic canister for 35 mm Kodak slide films baited with one mealworm and attached to the floor. In a previous study [8] six subjects (observers) were allowed to observe a conspecific model opening the plastic box in a peculiar way (mouth-opening) before they were tested individually. In a first test session of that study they were confronted with 15 half-shut canisters while in a second test session the 15 canisters were completely shut. Opening actions of these two sessions did not enter the motion analysis. In this study we tested the same observers for a third time as well as the model and 24 naïve animals (non-observers). For this purpose each of the observers and the model were separated in an experimental cage where they were confronted with one completely shut film canister. After the subjects had opened the canister and retrieved the mealworm, the canister was refilled and closed again. This process was continued until the subjects had opened 15 canisters or did not respond to the canister for 10 minutes. The test procedure for the non-observers differed in two ways from the procedure for the observers: In order to accustom the animals to the apparatus and to allow them to gather the same amount of experience as the observers could gather during their initial tests non-observers were confronted with 15 half-shut canisters prior to testing with completely shut canisters. And, if a subject failed to open the completely shut canister or did not manipulate it for 5 minutes three additional trials with half-shut canisters were interspersed in the test session to reinforce opening attempts.

### Video coding

For analysis the frame size of the video images was reduced to 520×390 pixel and all readings were taken at a precision level of one pixel. The images were calibrated with one pixel equaling 0.5 mm in the medial plane of the film canister. Each sequence was coded 10 times. For the calculation of the primary motion parameters (horizontal and vertical motion as well as rotation of the head between two consecutive frames) we calculated mean values for each trace point from the 10 repeated measurements. Single measures of a trace point which deviated from the mean for more than twice the standard deviation were regarded as unreliable measures and excluded. Trace points for which less than six reliable measures were recorded, were excluded from further analysis. To calculate the primary movement parameters it was required that at least three corresponding trace points were visible in all pairs of two consecutive frames. Sequences which did not fulfil this requirement were excluded from the sample in order to assure a high internal validity of the coding process. These stringent criteria reduced the dataset that entered the final analysis to 6 model sequences, 14 observer sequences and 21 non-observer sequences.

### Motion analysis

Mouth-opening actions were defined as successful when the subject removed the lid with its mouth and received the reward. Only successful opening actions of completely shut canisters were analyzed. The critical phase of the mouth-opening action was defined as the period between the point of time where the subjects' upper jaw connects with the lid of the canister and the point of time where the lid starts to move upwards. The head motion of the subjects was recorded with a digital video-camcorder (Sony DVX 1000) that was positioned 80 cm from the window with the lens at the same height as the lid of the film canister. The video sequences were analyzed with a MATLAB routine by manually identifying the position of five morphological features (trace-points) in the face of the subject on a frame-to-frame basis (25 frames per s). The coordinates of the trace-points were used to calculate horizontal- and vertical movement as well as rotation to fit the trace points of each frame to the trace points of the succeeding frame. Thereafter, we were able to calculate the following motion parameters: The inclination of the head defined as the angle between the axis from the subjects' canthus to the corner of the mouth and the vertical axis (1a) at the beginning of the sequence, and (1b) at the end of the sequence, (1c) the inclination change of the head by subtraction of the head inclination at the beginning of the sequence from the head inclination at the end of the sequence, (2) the overall motion direction defined as the direction of the vector from the central point of the head in the first frame of the sequence to the central point of the head in the last frame, (3) the total path-length, defined as the sum of the absolute length of the vectors from the central point of the head in each frame to the central point of the head in the consecutive frame, (4) the direct path defined as the absolute length of the vector from the central point of the head in the first frame of the sequence to the central point of the head in the last frame, and (5) a detour factor defined as the fraction of the total path length divided by the direct path length. As the inclination change of the head (1c) is a linear combination of parameters 1a and 1b (the inclination of the head at the beginning and at the end of the sequence), the latter were not included in the data analysis, hence reducing the number of parameters to five.

### Observer accuracy and reliability

To determine observer accuracy we analyzed the variance in the measurements of the single trace-points within the 10 repetitions by the coder BV. The mean standard deviation for the mean of the repeated measures was 0.53±0.13 mm in the horizontal and 0.51±0.15 mm in the vertical – which is approximately 1 pixel. To control for observer bias we asked eight independent raters to code two randomly selected movement sequences (one observer and one non-observer movement). These coders were not told to which experimental group the animals belonged, nor did they see sequences of the model prior to coding. Observer accuracy of the independent coders was with 0.63±0.23 mm in the horizontal and 0.64±0.19 mm in the vertical slightly lower as of coder BV – a result which we assume to be due to the higher amount of experience of coder BV. Comparing estimates of the horizontal and vertical movement parameters evaluated from the measurements made by coder BV and the independent coders we didn't find significant differences between sequences of observers and non-observers (MANOVA: F = 1.621; df = 2,132; P = 0.202; R^2^ = 0.024). Estimates of the movement parameters from the measurements by coder BV and the independent coders were very similar with a mean deviation of 0.26±0.16 mm for non-observer sequences and 0.21±0.15 mm for observer sequences.
